# Quantitative Analysis of Primary Compressive Trabeculae Distribution in the Proximal Femur of the Elderly

**DOI:** 10.1111/os.14141

**Published:** 2024-07-01

**Authors:** Cheng Xu, Hang Li, Chao Zhang, Feng Ge, Qing He, Hua Chen, Licheng Zhang, Xuedong Bai

**Affiliations:** ^1^ Department of Orthopedics The Sixth Medical Center of PLA General Hospital Beijing China; ^2^ Senior Department of Orthopedics The Fourth Medical Center of PLA General Hospital Beijing China; ^3^ Department of Hyperbaric Oxygen The Sixth Medical Center of PLA General Hospital Beijing China

**Keywords:** CT imaging, hip fracture, PCT, primary compressive trabeculae, proximal femur

## Abstract

**Objective:**

As osteoporosis progresses, the primary compressive trabeculae (PCT) in the proximal femur remains preserved and is deemed the principal load‐bearing structure that links the femoral head with the femoral neck. This study aims to elucidate the distribution patterns of PCT within the proximal femur in the elderly population, and to assess its implications for the development and optimization of internal fixation devices used in hip fracture surgeries.

**Methods:**

This is a retrospective cohort study conducted from March 2022 to April 2023. A total of 125 patients who underwent bilateral hip joint CT scans in our hospital were enrolled. CT data of the unaffected side of the hip were analyzed. Key parameters regarding the PCT distribution in the proximal femur were measured, including the femoral head's radius (R), the neck‐shaft angle (NSA), the angle between the PCT‐axis and the head–neck axis (α), the distance from the femoral head center to the PCT‐axis (δ), and the lengths of the PCT's bottom and top boundaries (L‐bottom and L‐top respectively). The impact of gender differences on PCT distribution patterns was also investigated. Student's *t*‐test or Mann–Whitney U test were used to compare continuous variables between genders. The relationship between various variables was investigated through Pearson's correlation analysis.

**Results:**

PCT was the most prominent bone structure within the femoral head. The average NSA, α, and δ were 126.85 ± 5.85°, 37.33 ± 4.23°, and 0.39 ± 1.22 mm, respectively, showing no significant gender differences (*p* > 0.05). Pearson's correlation analysis revealed strong correlations between α and NSA (*r* = −0.689, *p* < 0.001), and R and L‐top (*r* = 0.623, *p* < 0.001), with mild correlations observed between δ and NSA (*r* = −0.487, *p* < 0.001), and R and L‐bottom (*r* = 0.427, *p* < 0.001). Importantly, our study establishes a method to accurately localize PCT distribution in true anteroposterior (AP) radiographs of the hip joint, facilitating precise screw placement in proximal femur fixation procedures.

**Conclusion:**

Our study provided unprecedented insights into the distribution patterns of PCT in the proximal femur of the elderly population. The distribution of PCT in the proximal femur is predominantly influenced by anatomical and geometric factors, such as NSA and femoral head size, rather than demographic factors like gender. These insights have crucial implications for the design of internal fixation devices and surgical planning, offering objective guidance for the placement of screws in hip fracture treatments.

## Graphical abstract

A novel method for extracting the principal compressive trabeculae (PCT) from clinical CT scans of elderly hip joints was introduced. This study significantly advances our understanding by quantitatively describing the PCT distribution, and shedding light on the internal bone architecture of the proximal femur.

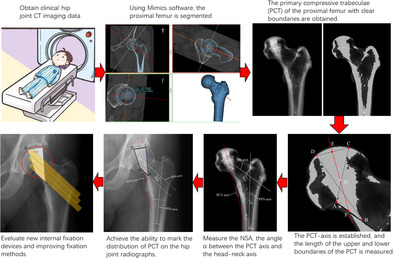

## Introduction

Elderly hip fracture is a worldwide public health concern, which causes significant morbidity, disability, mortality and socioeconomic pressure.^1^, ^2^ Despite the prevalent view that low bone mineral density (BMD) is a primary risk factor for fractures, numerous studies have highlighted the significant role of trabecular bone architecture in maintaining the biomechanical integrity of bone tissue.^3^, ^4^ Over recent decades, the trabecular bone within the proximal femur has emerged as a focal point of research, attracting substantial efforts to demystify its internal architecture and physiological loading patterns.^5^, ^6^, ^7^, ^8^, ^9^ Unlike cortical bone, trabecular bone is often celebrated for its self‐optimizing, trajectorial architecture.^10^, ^11^ As osteoporosis progresses, the bone mass within the proximal femoral trabeculae diminishes rapidly, particularly in structures that are less mechanically stimulated.^12^, ^13^ Nonetheless, the primary compressive trabeculae (PCT)—a dense column of trabecular bone extending from the superior aspect of the femoral head to the medial cortex of the femoral neck—remains relatively preserved and is deemed the principal load‐bearing structure that links the femoral head with the femoral neck.^6^, ^14^, ^15^, ^16^, ^17^, ^18^, ^19^ Consequently, exploring the PCT not only sheds light on the pathogenesis of hip fractures but also aids in refining the criteria for evaluating the effectiveness of implant selection.^20^, ^21^

Despite numerous attempts to map the distribution of PCT within the proximal femur, the conventional visual extraction method for identifying trabecular columns has been criticized for its substantial spatial deviation errors, casting doubts on its reliability.^8^, ^11^, ^14^, ^22^, ^23^, ^24^, ^25^ A significant challenge in assessing and quantifying PCT distribution lies in the unique nature of trabecular structures—no two are exactly alike. The PCT boundary is often irregular and challenging to define.^4^ Thus, identifying a reliable method to determine PCT distribution within the proximal femur holds great value.

This study introduces a novel approach, utilizing personalized threshold segmentation to extract the PCT from clinical computed tomography (CT) scans of elderly hip joints. We aim to clarify the following currently unclear questions: (i) What is the most prominent bone structure within the femoral head?; (ii) How to depict the PCT distribution within the proximal femur?; and (iii) What are the main factors determining the distribution of PCT?

## Methods

### Patients

This study received approval from the Medical Ethics Committee of the Chinese PLA General Hospital (approval number HZKY‐PJ‐2023‐17). Between March 2022 and April 2023, we conducted a retrospective review of patients aged 65 and older who underwent bilateral hip joint CT scans in our hospital due to hip joint pain resulting from accidental injuries. We selected the unaffected side of the hip for analysis. The exclusion criteria were: (i) bilateral hip fractures; (ii) previous hip surgeries; (iii) congenital deformities; (iv) femoral head necrosis or hip arthritis. The CT scans were performed using a Philips iCT 256 scanner (Amsterdam, the Netherlands) with consistent scanning parameters (120 kV; 216 mA; slice thickness 1.0 mm, interval 1.0 mm).

### Establishment of the Midcoronal Plane of the Proximal Femur

**FIGURE 1:**
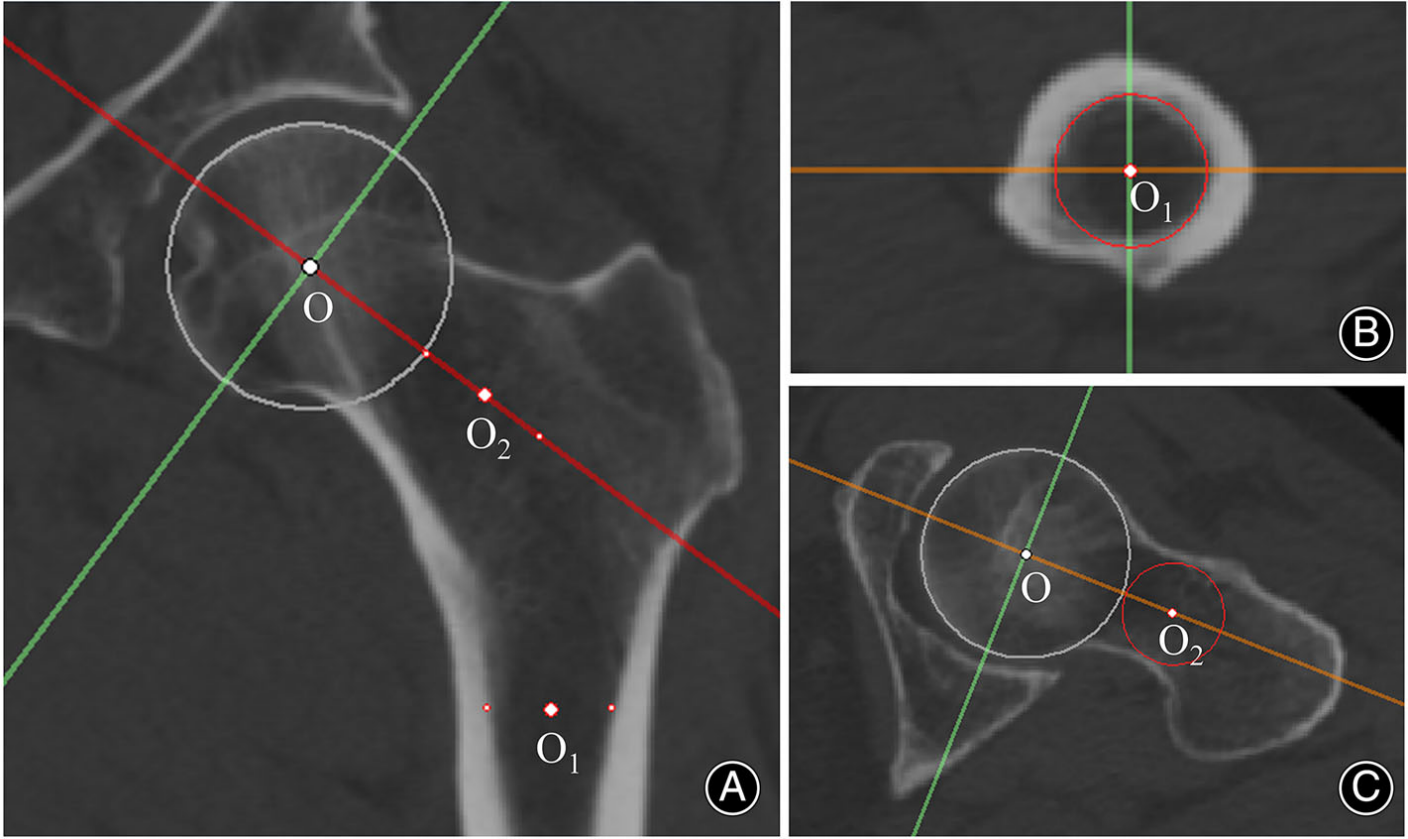
Establishment of the midcoronal plane of the proximal femur. A sphere was created to approximately match the articular surface of the femoral head, and the sphere center was located as Point O (A, C). In the axial sequence, the plane close to the lower edge of the lesser trochanter was located, in which a circle that best match the medullary cavity was created to locate the center point of the medullary cavity (Point O1) (B). The sequences were then resliced around Point O to obtain a midsagittal plane of the femoral neck, on which the center point of the femoral neck isthmus was determined by drawing a circle that was tangent to both the anterior and posterior cortexes (Point O2) (C). Finally, the standard midcoronal plane of the proximal femur which passes through Points O, O1 and O2, was obtained by re‐slicing the sequences around Point O (A).

Utilizing CT data in Digital Imaging and Communication in Medicine (DICOM) format, we analyzed images of the proximal femur with Mimics 21.0 software (Materialise, Leuven, Belgium). Initially, a sphere was constructed to approximate the articular surface of the femoral head.^26^ The center of this sphere, deemed the femoral head center, and its radius (R) were noted. By re‐slicing the image sequences centered around the femoral head, we established the midcoronal plane, which traverses the femoral head center, the midpoint of the femoral neck isthmus, and the center of the medullary cavity at the lesser trochanter's lower edge (Figure 1).

### Establishment of the Head–neck Axis (HN‐axis) and Measurement of Femoral Head's Mean Hounsfield Unit (HU) Value

**FIGURE 2:**
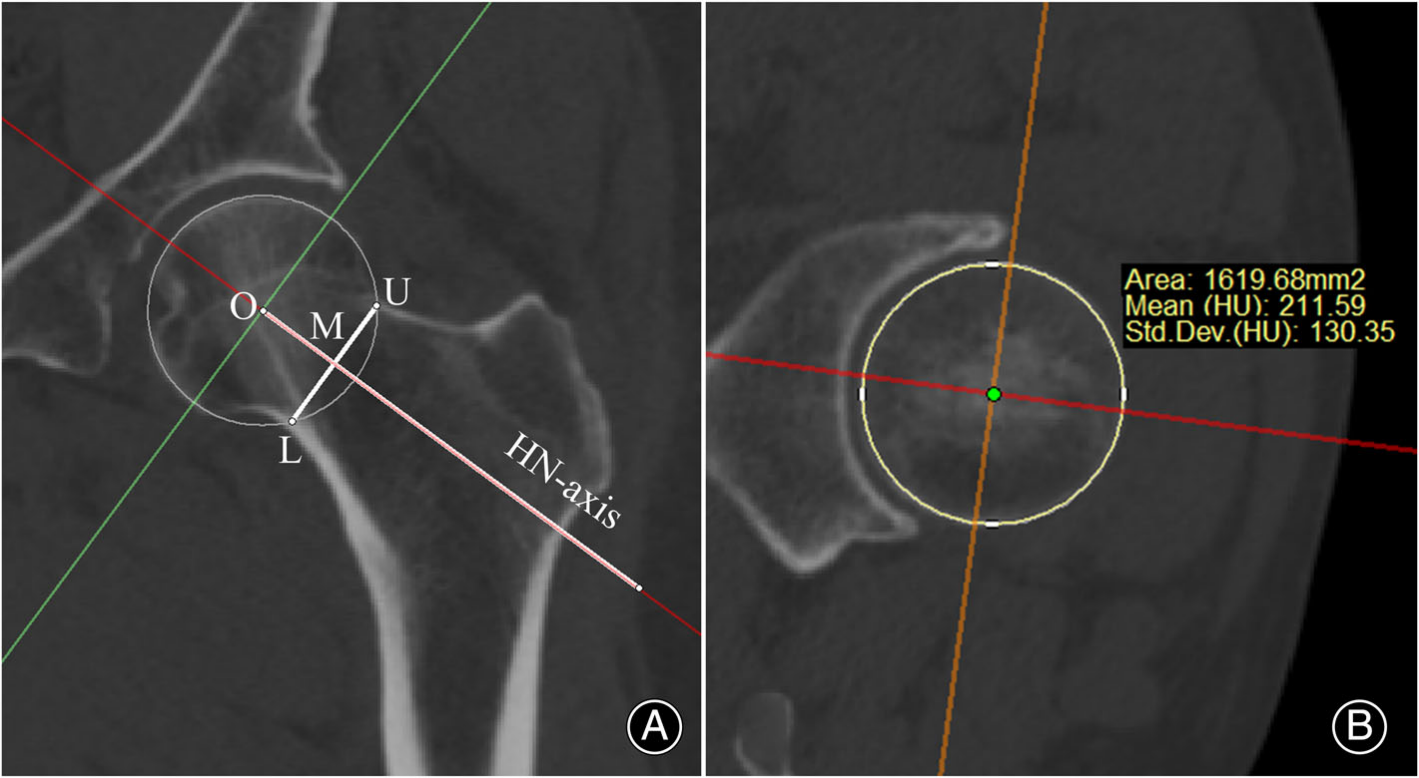
Establishment of the HN‐axis and measurement of the mean HU value of the femoral head. (A) shows the midcoronal plane of the proximal femur, on which the femoral head center was marked as Point O. The intersections of the sphere and the upper and lower inner cortex of the femoral neck were marked as Point U and L, respectively. The midpoint of line UL was located as Point M. Then line M was created as HN‐axis. (B) is the orthogonal plane of the midcoronal plane which passes through the femoral head center and is perpendicular to the HN‐axis. On this plane, the contour of the femoral head is always a circle, and the mean HU value of the femoral head can be measured standardly. In this case, the mean HU of the femoral head was 211.59 HU according to the measurement.

In the midcoronal plane, the HN‐axis was delineated by drawing a line through the center points of the femoral head and neck. In the axial sequence of the 3D coordinate system, we identified the plane passing through the femoral head center and perpendicular to the HN‐axis. On this plane, the femoral head contour appears circular, facilitating the measurement of the mean HU value of the femoral head (Figure 2).

### Segmentation of the Proximal Femur and Creation of Auxiliary Lines

**FIGURE 3:**
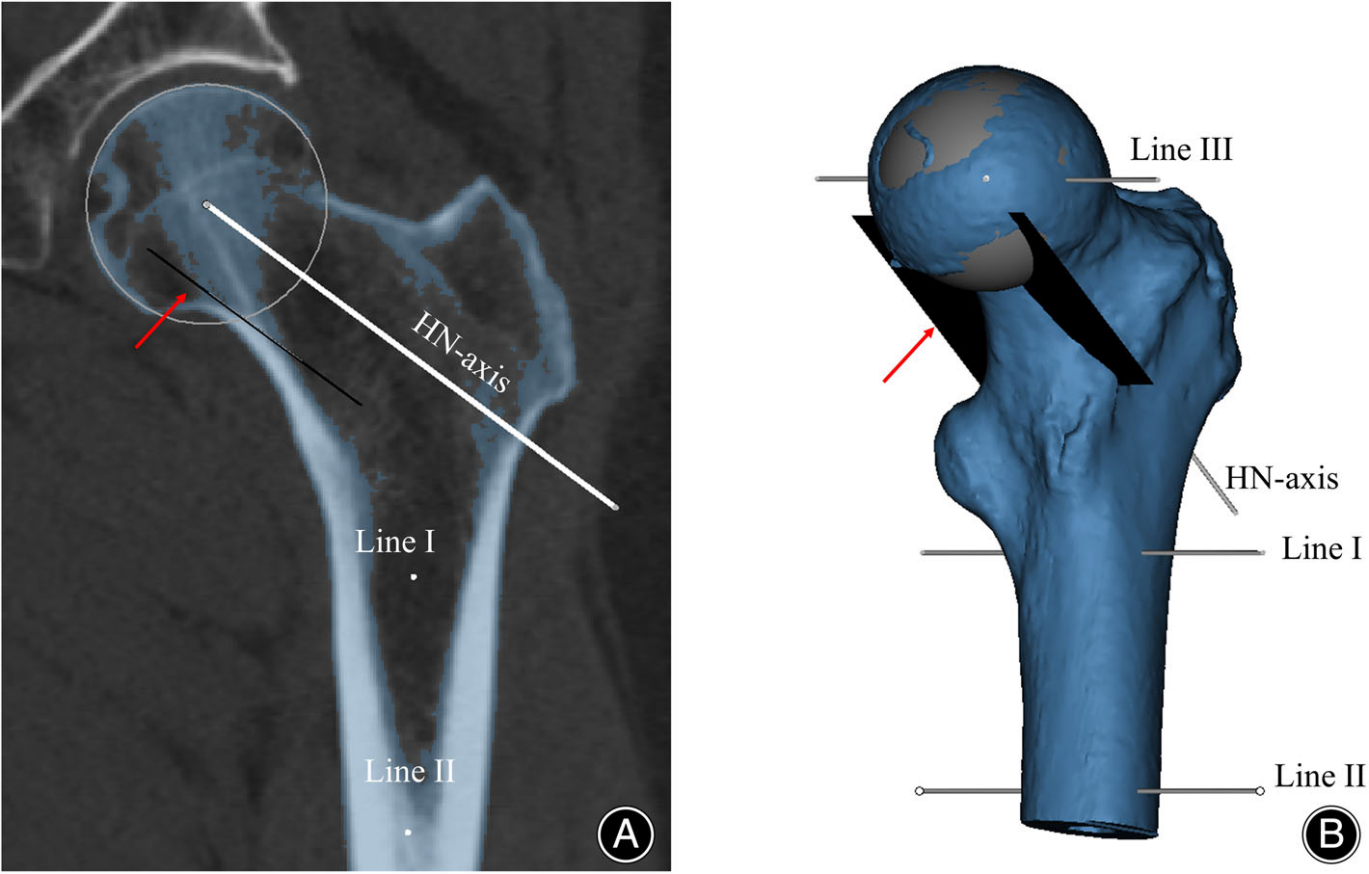
Segmentation of the proximal femur and creation of auxiliary lines. (A) The midcoronal plane of the proximal femur, which has been segmented from the pelvis and soft tissue through threshold segmentation, using the mean HU value of the femoral head to set the minimum threshold. (B) The 3D model of the proximal femur following segmentation. On the midcoronal plane, an orthogonal plane is established, tangent to the medial cortex of the proximal femur and parallel to the HN‐axis, aiding in the determination of the PCT's bottom boundary (indicated by arrows in a and b). For proximal femoral shaft axis calibration, two lines perpendicular to the midcoronal plane are drawn: Line I intersects the medullary cavity's center point at the lower edge of the lesser trochanter, and line II intersects the center point 5cm below the lesser trochanter. To locate the femoral head center, line III is drawn perpendicular to the midcoronal plane and passes through the center of the femoral head.

Threshold segmentation was employed for segmenting the proximal femur, setting the minimum threshold according to the femoral head's mean HU value. An orthogonal plane, tangent to the medial cortex of the proximal femur and parallel to the HN‐axis, aided in defining the PCT's bottom boundary. Three auxiliary lines, perpendicular to the midcoronal plane, were also established for subsequent steps (Figure 3).

### Coronal 2D Projection of the Proximal Femur

**FIGURE 4:**
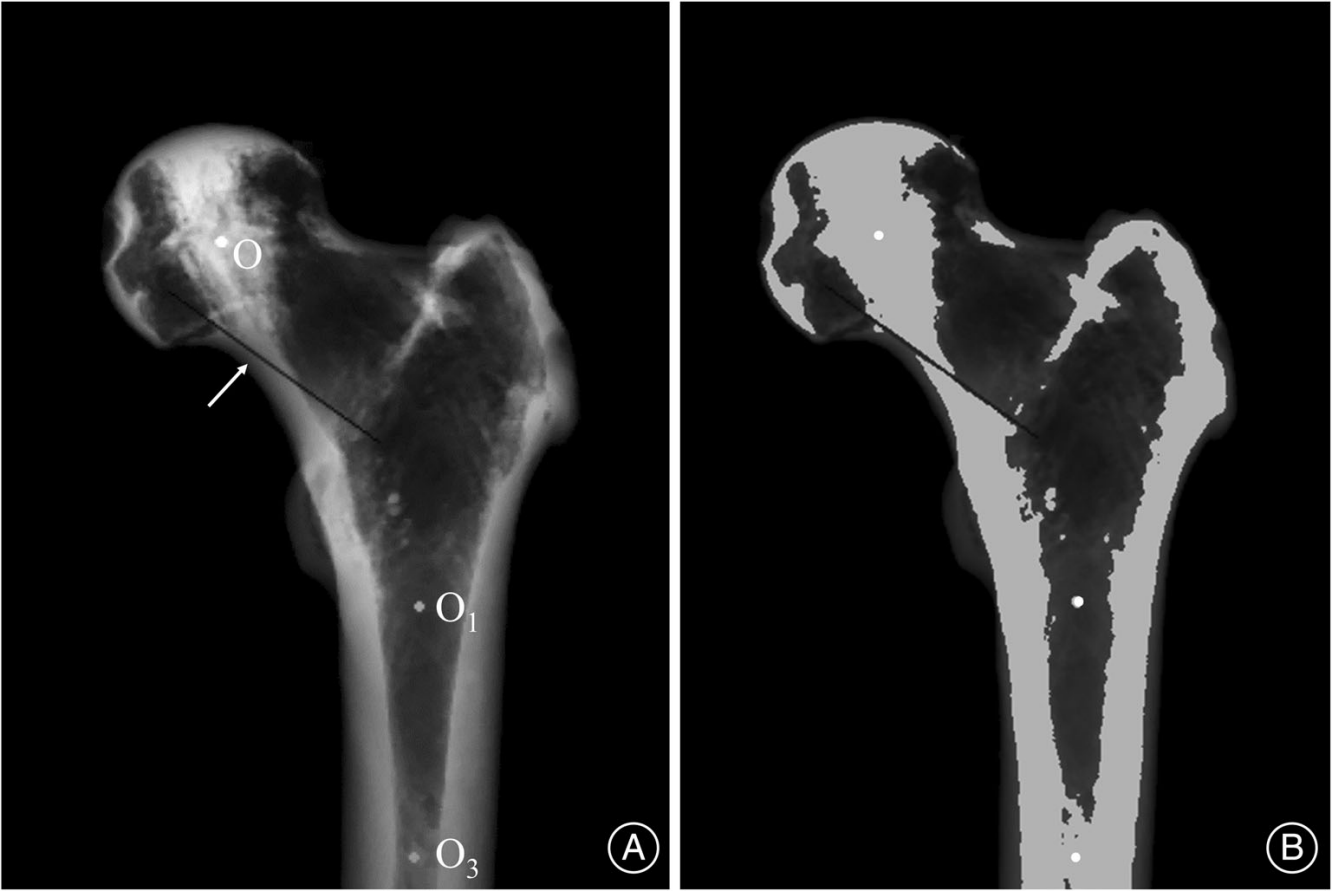
Coronal 2D projection of the proximal femur. (A) The standard coronal 2D projection of the proximal femur, derived from the 3D images. Within this 2D projection, the auxiliary plane is represented as a line (indicated by an arrow), whereas the previously established auxiliary lines are depicted as points (labeled as Points O, O1, and O3). (B) The binarized version of A, which has been processed through threshold segmentation to enhance contrast and clarity.

We projected the segmented 3D images of the proximal femur and auxiliary lines along the axis perpendicular to the midcoronal plane, generating a standard coronal 2D projection image (Figure 4A). The pixel value of this image was determined by summing the CT image data across layers, according to the formula: $\text{Image}\left(x,y\right)=\sum_{i=1,2,3\ldots n}\mathrm{CT}\left(\mathrm{xi},\mathrm{yi}\right)$, where *i* represents the number of layers. To delineate the PCT boundary clearly, further segmentation and binarization were performed using threshold segmentation based on the femoral head's mean HU value (Figure 4B).

### Measurements of the PCT on the Coronal Projection

**FIGURE 5:**
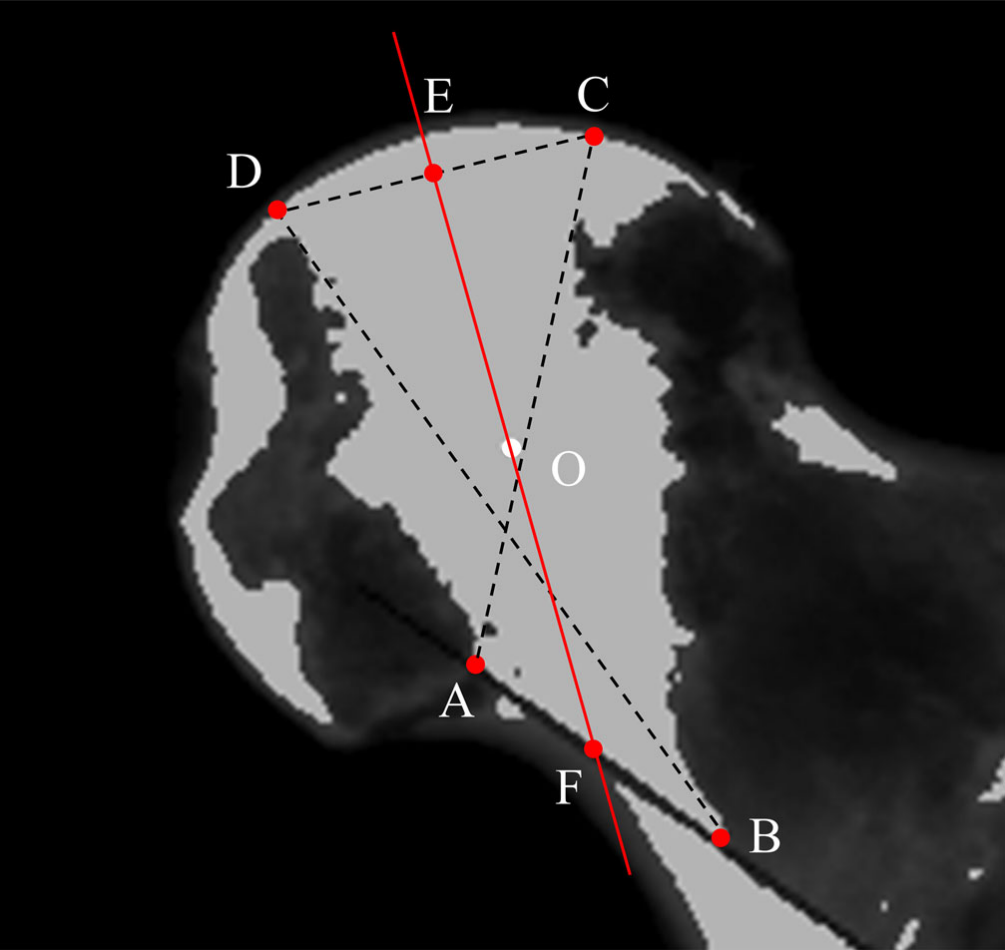
The method for assessing the quadrilateral structure of the PCT. On the coronal 2D projection of the proximal femur, the bottom boundary of the PCT is defined by the intersection of the PCT structure with the tangent line at the inferior aspect of the femoral neck (line AB). Then the top boundary of the PCT (line CD) is determined by line AC and line BD which are tangent to the lateral and medial edge of the PCT structure, respectively. The PCT‐axis is determined by drawing line EF to connect the midpoints of the top (line CD) and bottom (line AB) boundaries. The perpendicular distance from the center of the femoral head (Point O) to the PCT‐axis (line EF) is measured and documented as δ. Additionally, the lengths of the bottom (line AB) and top (line CD) boundaries of the PCT are measured and recorded as L‐bottom and L‐top, respectively. These measurements are critical for understanding the structural integrity and stress transduction pathways of the proximal femur.

**FIGURE 6:**
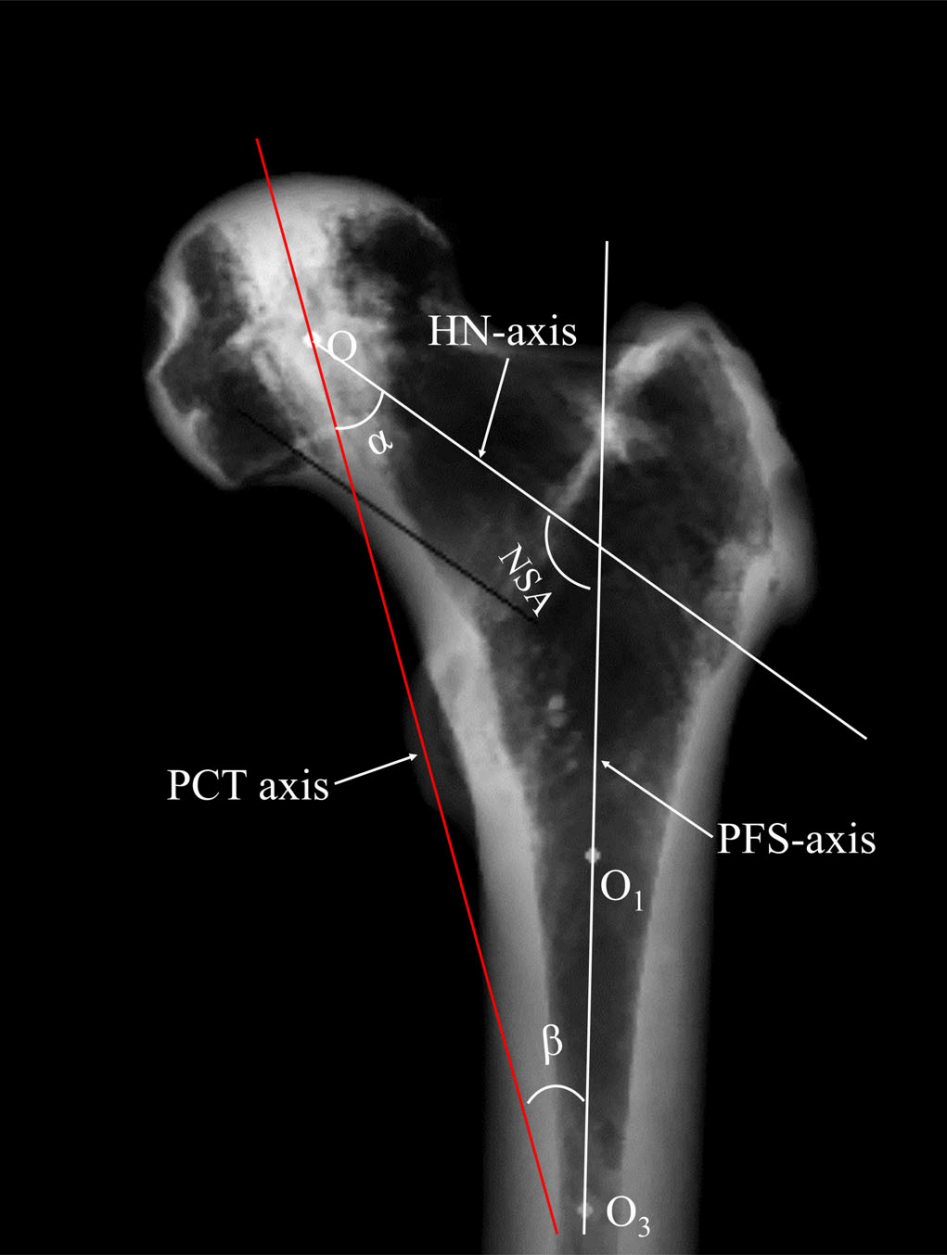
Measurement of angles related to PCT‐axis. Points O1 and O3 represent the central points of the medullary cavity as determined by the coronal projection. The PFS‐axis is defined by the line connecting Points O1 and O3. The NSA is the angle created by the intersection of the HN‐axis with the PFS‐axis. The angle α is defined by the intersection of the PCT‐axis with the HN‐axis. The angle β, formed by the intersection of the PCT‐axis with the PFS‐axis, can be calculated using the formula β = 180° –NSA – α.

Viewing the PCT's coronal projection as a structurally integral quadrilateral, essential for conducting compressive stress from the femoral head to the neck's medial cortex, we established the PCT‐axis by linking the midpoints of its bottom and top boundaries. The distance from the femoral head center to the PCT‐axis (δ) was measured, with δ assigned a positive value if the PCT‐axis was lateral to the femoral head center, and negative otherwise. The lengths of the PCT's bottom and top boundaries were recorded as L‐bottom and L‐top, respectively (Figure 5). The proximal femoral shaft (PFS)‐axis was determined by the projected central points of the medullary cavity, allowing measurement of the proximal femur's neck‐shaft angle (NSA) formed by the HN‐axis and PFS‐axis. The angles between the PCT‐axis and the HN‐axis (α), and the PCT‐axis and PFS‐axis (β), were measured, with β calculated as β = 180°—NSA—α (Figure 6).

### Statistical Analysis

To assess the distribution of the collected data, we initially applied the Kolmogorov–Smirnov test. Based on the distribution characteristics, data adhering to a normal distribution were analyzed using Student's *t*‐test, while those not conforming to normal distribution criteria were evaluated with the Mann–Whitney U test. The relationship between various variables was investigated through Pearson's correlation analysis, providing a quantitative measure of the degree of association between them. All statistical procedures were conducted utilizing SPSS version 20 software (SPSS Inc., Chicago, IL, USA). Statistical significance was set at *p* < 0.05.

The reliability of the measurement techniques employed in this study was gauged using the intra‐class correlation coefficient (ICC). To ascertain both intra‐ and inter‐observer reliability, two authors independently performed measurements on a randomly selected subset of 20 samples. These measurements were repeated twice, with a four‐week interval between sessions, to minimize recall bias and ensure the stability of the measurement process over time.

## Results

### Study Population

Our study meticulously selected 125 elderly patients, comprising 41 men and 84 women, with 55 involving the right hip and 70 the left hip. This selection was made in strict adherence to our inclusion and exclusion criteria. The mean age of the patients was 80.26 ± 8.10 years, with ages ranging from 65 to 97 years. Notably, the analysis revealed no significant age difference between male and female participants.

### PCT Analysis

**FIGURE 7:**
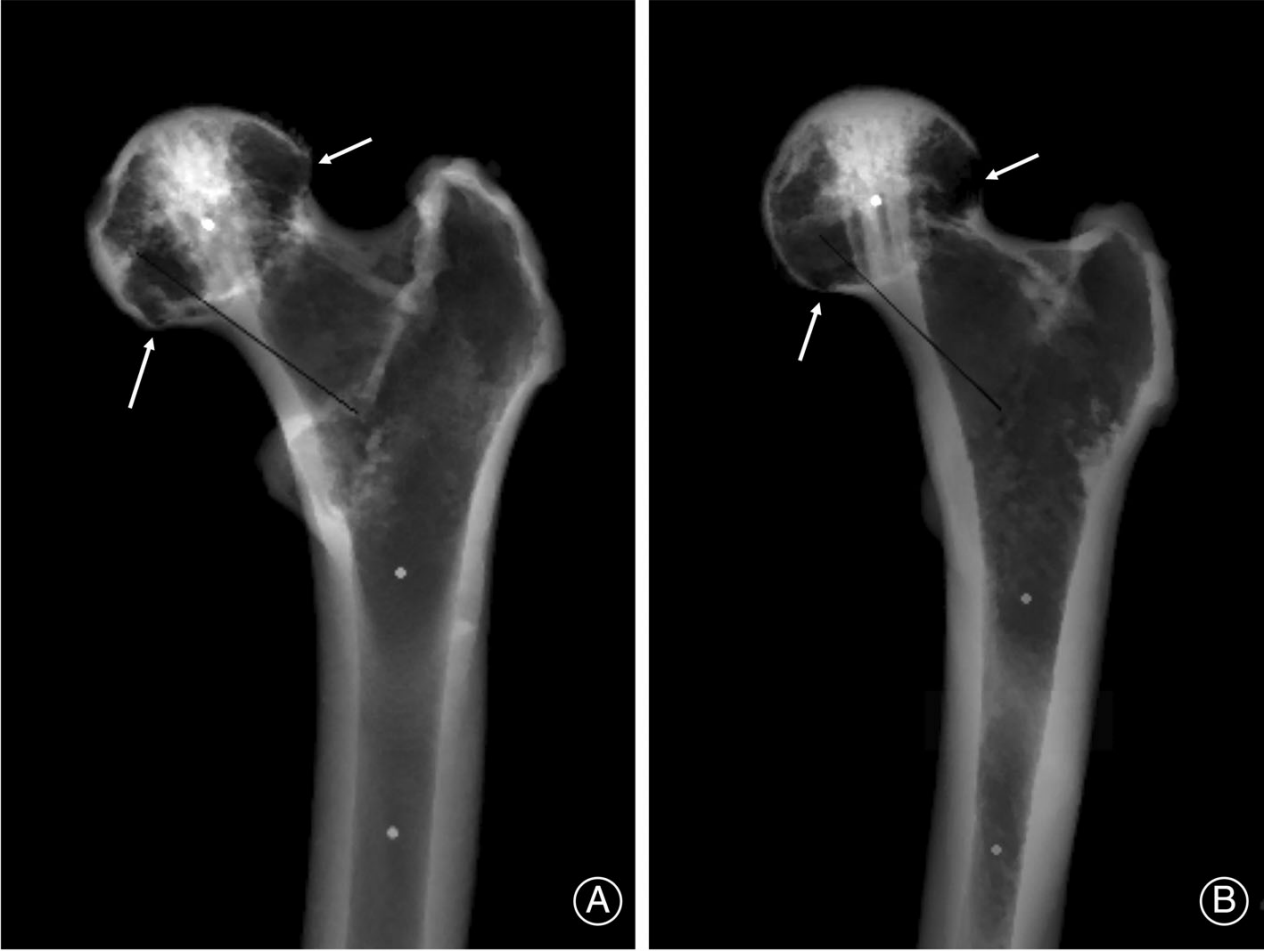
Representative cases after segmentation and coronal projection. In the femoral head, the PCT is always the most prominent bone structure. One the contrary, there are always weak or defect areas in the femoral head cortex, especially in the junction area between the femoral head and neck. (A) Arrows show weak areas. (B) Arrows show defect areas.

Upon conducting threshold segmentation and coronal projection of the proximal femur, we consistently identified the PCT as the most pronounced bone structure within the femoral head across all subjects. However, it was observed that the cortex of the femoral head exhibited areas of weakness or defects, particularly notable at the junctions between the femoral head and neck (Figure 7).

### Measurement Outcomes

The parameter measurement results were summarized in Table 1.

**TABLE 1:** The main parameter measurements of the proximal femur.

Items	Men (*n* = 41)		Women (*n* = 84)		*p*‐value	Total (*n* = 125)	
	Mean ± SD	Range	Mean ± SD	Range		Mean ± SD	Range
R (mm)	23.91 ± 1.22	21.40–27.28	21.43 ± 1.14	18.16–24.85	<0.001	22.25 ± 1.65	18.16–27.28
Mean HU value (Hu)	178.46 ± 53.25	102–308	136.93 ± 38.85	66–271	<0.001	150.55 ± 48.05	66–308
NSA (°)	126.95 ± 5.76	115.07–139.40	126.79 ± 5.93	112.70–143.06	0.885	126.85 ± 5.85	112.7–143.06
α (°)	37.12 ± 4.01	29.87–46.22	37.44 ± 4.36	27.06–48.60	0.693	37.33 ± 4.23	27.06–48.60
β (°)	15.93 ± 4.39	3.44–25.49	15.77 ± 4.20	5.78–24.91	0.847	15.82 ± 4.25	3.44–25.49
δ (mm)	0.30 ± 1.27	(−2.36)–2.92	0.44 ± 1.20	(−2.69)–3.38	0.564	0.39 ± 1.22	(−2.69)–3.38
L‐bottom (mm)	19.86 ± 3.39	12.97–26.00	18.02 ± 2.75	10.97–29.45	0.002	18.62 ± 3.08	10.97–29.45
L‐top (mm)	23.63 ± 3.27	17.68–31.00	20.26 ± 2.99	15.13–29.89	<0.001	21.37 ± 3.46	15.13–31.00

#### Radius (R)

The average femoral head radius was 22.25 ± 1.65 mm, with a significant gender disparity noted (men: 23.91 ± 1.22 mm *vs* women: 21.43 ± 1.14 mm).

#### Hounsfield Unit (HU) Value

We observed a broad variance in the mean HU value of the femoral head (66 to 308 Hu), with significant differences between genders (men: 178.46 ± 53.25 HU *vs* Women: 136.93 ± 38.85 HU).

#### Angles and Lengths

The average NSA, α, and β were 126.85 ± 5.85°, 37.33 ± 4.23°, and 15.82 ± 4.25°, respectively, showing no significant gender differences. Similarly, the average δ showed no significant gender difference at 0.39 ± 1.22 mm. However, significant gender differences were observed in the average L‐bottom and L‐top measurements (19.86 ± 3.39 mm *vs* 18.02 ± 2.75 mm and 23.63 ± 3.27 mm *vs* 20.26 ± 2.99 mm, respectively).

### Reliability and Correlation Analysis

The intra‐class correlation coefficient (ICC) confirmed good inter‐ and intra‐observer reliability across all parameter measurements (Table 2). Pearson's correlation analysis revealed strong correlations between α and NSA (*r* = −0.689, *p* < 0.001), and β and NSA (*r* = −0.691, *p* < 0.001), with mild correlations observed between δ and NSA (*r* = −0.487, *p* < 0.001). Linear regression analyses were applied with the NSA set as the independent variables, α, β or δ as the dependent variables. Then α = 100.51–0.498 × NSA (*R* ^2^ = 0.474), β = 79.49–0.50 × NSA (*R* ^2^ = 0.478), δ = 13.262–0.101 × NSA (*R* ^2^ = 0.237). Strong correlations were also found between R and L‐top (*r* = 0.623, *p* < 0.001), while moderate correlations were found between R and L‐bottom (*r* = 0.427, *p* < 0.001). Linear regression analyses were applied with the R set as the independent variables, L‐top or L‐bottom as the dependent variables. Then L‐top = −7.695 + 1.306 × R (*R* ^2^ = 0.388), L‐bottom = 0.839 + 0.799 × R (*R* ^2^ = 0.182).

**TABLE 2:** Reliability study results.

Items	Intra‐observer		Inter‐observer	
	ICC	95% CI	ICC	95% CI
*R* (mm)	0.982	0.955–0.993	0.980	0.951–0.992
Mean HU value (Hu)	0.997	0.992–0.999	0.995	0.989–0.998
NSA (°)	0.966	0.916–0.986	0.964	0.914–0.986
α (°)	0.897	0.763–0.985	0.911	0.793–0.964
δ (mm)	0.928	0.828–0.971	0.877	0.721–0.949
L‐bottom (mm)	0.942	0.862–0.977	0.924	0.821–0.969
L‐top (mm)	0.923	0.813–0.869	0.883	0.729–0.952

### PCT Localization in Anteroposterior (AP) Radiographs

**FIGURE 8:**
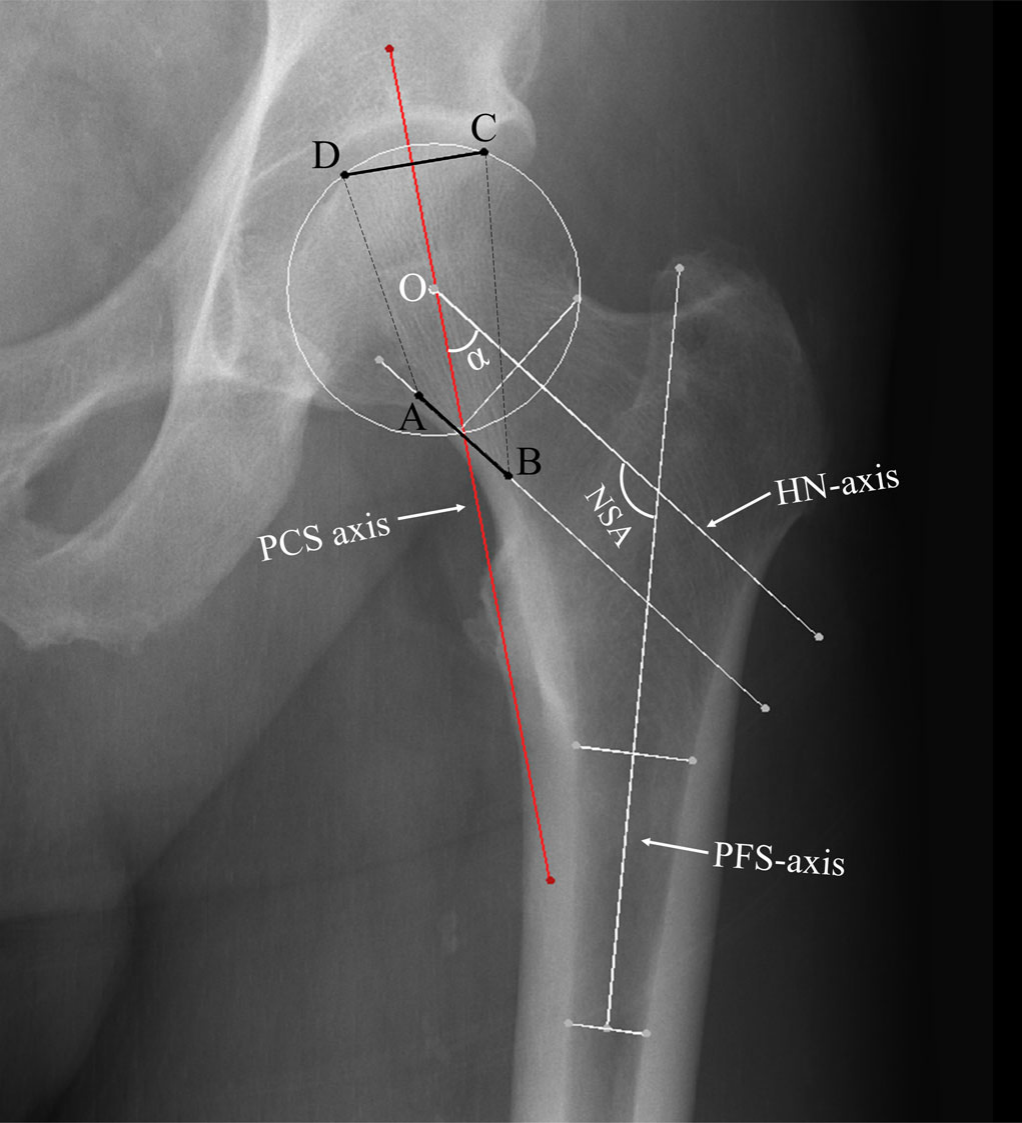
The method for identifying the location of the PCT on an AP radiograph of the hip joint. An analytical circle is drawn to approximate the contour of the femoral head's articular surface, with its center marked as Point O. The radius of this circle, denoted as R, is measured. The NSA is defined by the angle between the HN‐axis and the PFS‐axis, which are delineated using established methods. In this example, R is 26.51 mm, and the NSA is 126.68°. Applying our regression models, we calculate α as 37.42°, δ as 0.47 mm, L‐bottom as 22.02 mm, and L‐top as 26.93 mm. The PCT‐axis is then constructed by drawing a line 0.47 mm lateral to Point O at an angle of 37.42° with the HN‐axis. A line parallel to the HN‐axis and tangent to the medial cortex of the femoral neck is drawn to determine the bottom boundary of the PCT (line AB), which has a length of 22.02 mm and is bisected by the PCT‐axis. Similarly, the top boundary of the PCT (line CD) is identified by drawing a chord measuring 26.93 mm in length that is equally divided by the PCT axis. The quadrilateral ABCD thus formed outlines the distribution of the PCT on the radiograph.

The true AP radiograph of the hip joint is equivalent to the coronal projection of the proximal femur. Through our comprehensive measurements and analysis, we determined that the distribution of PCT can be accurately localized in any true AP radiograph of the hip joint (Figure 8). This finding underscores the potential for utilizing AP radiographs in the clinical assessment of PCT distribution within the proximal femur.

## Discussion

Our study provided a novel standard approach to extract the PCT from clinical CT scans of elderly hip joints. We confirmed that the PCT is the most prominent bone structure within the femoral head and should be the optimal fixation target for screws. We further quantitatively analyzed the distribution of PCT in the proximal femur and explored the factors determining the distribution of PCT. Finally, we established a standard method to localize PCT distribution in true AP radiographs of the hip joint, which has significant implications for screw placement in proximal femur fixation procedures.

### Extract the PCT from Clinical CT Scans of Elderly Hip Joints

In our study, we measured the mean HU value of each femoral head on a specifically determined plane, which was defined by the center of the femoral head, the midcoronal plane, and the HN‐axis. This approach allowed us to segment the proximal femur based on the mean HU value of the femoral head. Despite the wide variability in mean HU values among individuals (ranging from 66 to 308 HU), our segmentation method ensured that a continuous and complete PCT was extracted in every case. This method aligns with Stiehl *et al*.'s study, which visualized the gross anatomical structure of the PCT by removing the softer cancellous bone in the proximal femur, effectively mirroring our threshold segmentation approach but through a digital process.^18^

### The most Prominent Bone Structure within the Femoral Head

To achieve a standard AP projection of the proximal femur, we located the midcoronal plane within CT image sequences. This plane was determined by the center points of the femoral head, the femoral neck isthmus, and the medullary cavity at the lower edge of the lesser trochanter. The projection of the proximal femur was then aligned perpendicularly to this midcoronal plane. Consistent with previous findings, we observed areas of weakened cortex within the femoral head, particularly at the junctions between the femoral head and neck.^12^, ^15^, ^27^ However, following threshold segmentation and coronal projection, the PCT consistently emerged as the most prominent bone structure within the femoral head, reinforcing the notion that PCT serves as the primary load‐bearing structure and an optimal fixation target for screws.

### Depiction of PCT Distribution within the Proximal Femur

The structural optimization of trabecular bone to support and transfer loads is a well‐recognized phenomenon.^4^, ^28^ The trabeculae in the cancellous bone were influenced by the magnitude of the principal stress trajectory.^29^, ^30^ The PCT of the proximal femur represents a response to long‐term compressive stress, primarily borne by axially aligned trabeculae.^14^, ^31^, ^32^ Within the PCT, irregularly enlarged structures, likely epiphyseal scars or remnants of the proximal tension trabecular system, are occasionally observed.^11^, ^18^ Given the significant individual variability of these structures and their minimal impact on compressive stress transfer, our analysis disregarded their influence on the overall PCT structure. Consequently, we conceptualized the coronal projection of PCT as a quadrilateral bone plate facilitating the transfer of compressive stress from the femoral head's superior aspect to the medial cortex of the femoral neck.

Determining the PCT's boundaries involved establishing a plane in resliced 3D CT images, parallel to the HN‐axis, tangent to the femoral neck's medial cortex in the midcoronal plane, and perpendicular to the midcoronal plane. This method effectively identified the bottom boundary of the PCT, with the top boundary inversely determined based on the stress conduction mechanism. The PCT‐axis was then defined by connecting the midpoints of these boundaries.

### Factors Determining the Distribution of PCT

Our study also established a reference system for calibrating the PCT distribution within the proximal femur, utilizing the femoral head center, the HN‐axis, and the PFS‐axis as references. Despite significant anatomical variations among individuals, our analysis revealed close correlations between certain parameters (α, β, δ) and the neck‐shaft angle (NSA), as well as between the bottom and top lengths of the PCT (L‐bottom and L‐top) and the radius of the femoral head (R). These associations highlight the necessity of considering these anatomical parameters when planning surgical approaches and internal fixation strategies. Currently, lag screw cutout due to improper placement remains one of the major causes of proximal femoral internal fixation failure.^33^ Therefore, the ability to accurately localize the distribution of PCT in true AP radiographs of the hip joint represents a significant advancement. Our findings enable clinicians to utilize standard imaging techniques to identify the optimal regions for screw placement, thereby improving the precision and effectiveness of proximal femur fixation.

While previous research highlighted gender differences in proximal femur remodeling,^34^ our study found no significant gender differences in the distribution of the PCT‐axis. However, gender differences were noted in L‐bottom and L‐top measurements, which we attribute to bone size differences rather than inherent gender disparities. After standardizing these measurements using the femoral head radius (R), no significant gender differences were observed, underscoring that PCT distribution is primarily dependent on the NSA and femoral head size.

### Strengths and Limitations

Our study provided unprecedented insights into the distribution patterns of PCT in the proximal femur of the elderly population, marking a significant advancement in our understanding of proximal femoral bone architecture. However, several limitations should be acknowledged. First, our study was conducted using a specific demographic group of elderly patients, which may not represent the variations seen in younger populations or those with different ethnic backgrounds. Second, the CT scans were performed without randomization, and the side of the hip might influence the density and distribution of trabeculae due to dominance or pre‐existing conditions. This could introduce a bias in assessing the structural characteristics of the proximal femur. Third, our study design was cross‐sectional, which restricts our ability to infer causality or the progression of changes in PCT distribution over time. Longitudinal studies would be necessary to understand the dynamics of PCT changes with aging or following therapeutic interventions.

### Prospects of Clinical Application

Our study might provide an anatomical foundation for finite element analysis, biomechanical research, and the investigation of injury mechanisms in elderly hip fractures. In terms of direct clinical application, it offers valuable reference points for optimizing the design of fixation devices and assists surgeons in formulating individualized surgical plans, thereby enhancing surgical precision and improving treatment outcomes.

Nevertheless, our research is fundamentally based on imaging‐derived anatomical morphology, and its accuracy may be constrained by current technological capabilities. The reliability of our findings still requires validation through biomechanical and clinical studies.

## Conclusion

In conclusion, our study quantitatively investigated the distribution pattern of PCT in the proximal femur based on imaging anatomy of the elderly population. These findings hold significant reference value for the development of new internal fixation devices for hip fractures and provide objective guidance for the placement of various screws in proximal femur fixation. Based on our analysis, the following conclusions can be drawn: (i) for the elderly, the PCT is the most prominent bone structure connecting the femoral head and neck, making it an optimal fixation target for screws; (ii) the PCT within the proximal femur can be effectively extracted and calibrated through the standard methods provided in this study; (iii) the distribution of PCT is closely related to the NSA and the size of the femoral head, rather than gender; and (iv) in true AP radiographs of the hip joint representing the coronal projection of the proximal femur, the distribution of PCT can be largely localized based on our research results.

## Conflict of Interest Statement

All authors listed meet the authorship criteria according to the latest guidelines of the International Committee of Medical Journal Editors, and all authors are in agreement with the manuscript.

## Ethical Statement

This study has been approved by the Medical Ethics Committee of General Hospital of PLA (number HZKY‐PJ‐2023‐17).

## Author Contributions

XB conceived the study and wrote the manuscript. CX, HL, and CZ performed the study. FG, QH, HC, and LZ contributed to the data collection and interpretation of the results. All authors read and approved the final manuscript and consented to publish this manuscript.
